# Clinical Notes

**Published:** 1887-02

**Authors:** E. W. Berridge

**Affiliations:** London


### CLINICAL NOTES.

### E. W. Berridge, M. D., London.

A lady patient sends me the following case: Miss A. had white patches (like the eggs of flies) on both tonsils, extending thence to back of throat; tonsils enlarged and deep red; felt she would suffocate at night from full feeling in throat which prevented sleep; swallowing toast gave some pain, but seemed to clear the throat; drinking anything caused more pain in throat, and she had to gulp it down. *Lac caninum*$^{30}$ quickly cured.

I have recently cured with one dose of Fincke's millionth potency the following mental symptoms: "Omits the final letter or letters of a word when writing; mentions in speaking the object seen instead of the object thought of."

As I am arranging *Lac caninum* for the I. H. A., I shall be glad to receive any additional symptoms.

